# Real-time PCR quantification of spliced X-box binding protein 1 (XBP1) using a universal primer method

**DOI:** 10.1371/journal.pone.0219978

**Published:** 2019-07-22

**Authors:** Seung-Bin Yoon, Young-Ho Park, Seon-A Choi, Hae-Jun Yang, Pil-Soo Jeong, Jae-Jin Cha, Sanghoon Lee, Seung Hwan Lee, Jong-Hee Lee, Bo-Woong Sim, Bon-Sang Koo, Sang-Je Park, Youngjeon Lee, Young-Hyun Kim, Jung Joo Hong, Ji-Su Kim, Yeung Bae Jin, Jae-Won Huh, Sang-Rae Lee, Bong-Seok Song, Sun-Uk Kim

**Affiliations:** 1 National Primate Research Center, Korea Research Institute of Bioscience and Biotechnology, Chungcheongbuk-do, Republic of Korea; 2 Futuristic Animal Resource & Research Center, Korea Research Institute of Bioscience and Biotechnology, Chungcheongbuk-do, Republic of Korea; 3 Primate Resource Center, Korea Research Institute of Bioscience and Biotechnology, Jeollabuk-do, Republic of Korea; 4 Department of Functional Genomics, University of Science and Technology, Daejeon, Republic of Korea; University of Wisconsin-Milwaukee, UNITED STATES

## Abstract

X-box binding protein 1 (XBP1) mRNA processing plays a crucial role in the unfolded protein response (UPR), which is activated in response to endoplasmic reticulum (ER) stress. Upon accumulation of the UPR-converted XBP1 mRNA splicing from an unspliced (u) XBP1 (inactive) isoform to the spliced (s) XBP1 (active) isoform, inositol-requiring enzyme 1 α (IRE1α) removes a 26-nucleotide intron from uXBP1 mRNA. Recent studies have reported the assessment of ER stress by examining the ratio of sXBP1 to uXBP1 mRNA (s/uXBP1 ratio) via densitometric analysis of PCR bands relative to increased levels of sXBP1 to uXBP1 using a housekeeping gene for normalization. However, this measurement is visualized by gel electrophoresis, making it very difficult to quantify differences between the two XBP1 bands and complicating data interpretation. Moreover, most commonly used housekeeping genes display an unacceptably high variable expression pattern of the s/uXBP1 ratio under different experimental conditions, such as various phases of development and different cell types, limiting their use as internal controls. For a more quantitative determination of XBP1 splicing activity, we measured the expression levels of total XBP1 (tXBP1: common region of s/uXBP1) and sXBP1 via real-time PCR using specific primer sets. We also designed universal real-time PCR primer sets capable of amplifying a portion of each u/s/tXBP1 mRNA that is highly conserved in eukaryotes, including humans, monkeys, cows, pigs, and mice. Therefore, we provide a more convenient and easily approachable quantitative real-time PCR method that can be used in various research fields to assess ER stress.

## Introduction

The endoplasmic reticulum (ER) is composed of two structures: a rough ER, with ribosomes attached to it, and a smooth ER (without ribosomes) [1]. Intracellular proteins are transformed from RNA (mRNA) to protein in the ER by post-translational modifications; folding and assembly, glycation, and disulfide bond formation are involved in the process, resulting in an active protein structure [2, 3]. The accumulation of unfolded or folded proteins in the ER triggers a transcriptional induction pathway, such as the unfolded protein response (UPR) [1, 3]. The UPR is a complex signaling system that increases the protein folding ability of the ER while reducing protein influx or decomposing an unfolded protein to reduce stress [3]. However, if this mechanism is not sufficient to restore homeostasis in the ER, the cell will undergo apoptosis, leading to death [4]. This process is mediated by the sensor proteins inositol-requiring endonuclease (IRE1), activating transcription factor 6 (ATF6) and PKR-like ER kinase (PERK) [3, 4]. The function of the UPR can be classified into gene expression related to ER function through an increase in transcription, termination of synthesis of the new protein by inhibiting protein translation, and degradation of unfolded proteins via ER-associated degradation (ERAD) [5]. Several gene analyses have shown that the UPR regulates genes involved mainly in protein transfer, folding, glycation, proteolysis, lipid biosynthesis, vesicle transport, and oxidation-reduction metabolism in the ER [6, 7].

IRE1/XBP1 is the most conserved UPR signaling mechanism among eukaryotes [8]. IRE1 is a Ser/Thr protein kinase and an endoribonuclease. When IRE1α is autophosphorylated by an increase in unfolded proteins in the ER, splicing occurs in the cytoplasm by IRE1α, which recognizes the secondary structure of XBP1 mRNA [3–5]. IRE1α cleaves a 26-bp intron in XBP1 mRNA, which changes the open reading frame to express a new C-terminal XBP1s protein. XBP1s increases the transcription of ERAD-related genes that degrade unfolded proteins, along with chaperone molecules that increase the folding of proteins such as transcription factors with the bZIP domain [4, 5, 9].

Conversion of unspliced XBP1 (uXBP1) to the spliced form (sXBP1) at the mRNA level was detected on agarose gels by semi-qPCR using a primer containing the cleavage site in the XBP1 gene. However, the difference of only 26 bp could not be clearly distinguished [8]. Therefore, a new method was developed to detect only sXBP1 using the restriction enzyme *Pst* I (cleavage site: CTG CAG) [10]. However, as this method also requires a preprocessing step, a qPCR method was recently developed to measure XBP1 in human cell lines [11]. In addition, transgenic mice have recently been generated to allow monitoring of XBP1 expression [12].

ER stress affects the function and differentiation of secretory cells and is related to lipid metabolism. ER stress is also involved in regulation of the immune response and causes many degenerative diseases [13]. In this study, we developed a universal primer targeting s/uXBP1 that can be used in humans, monkeys, cows, pigs, and mice. In cases in which the genes cannot be normalized to a housekeeping gene, such as cells in early embryonic development, differentiation, and solid cancer [14–16], we propose a method of quantifying total XBP1 (tXBP1) expression using an internal control to efficiently detect ER stress.

## Materials and methods

### Ethics statement

All procedures and use of all animals were approved by the Korea Research Institute of Bioscience and Biotechnology (KRIBB) Institutional Animal Care and Use Committee (Approval No. KRIBB-ACE-18130, KRIBB-ACE-18144). Cynomolgus maintained food at 20–25°C, humidity 40–50%, day and night adjustments 12 hours, feeding 3 times a day. Feeding mainly used primate-specific feeds, and seasonal fruits were fed as snacks. Various tools such as mirrors, balls and ladders are used for welfare of experimental animals. Experiment using mini-pig and mice were performed in accordance with the Guide for the care and use of laboratory Animals published by the National Institutes of Health. In order to reduce the pain of experimental animals, ketamine (5–10 mg/kg, IV) was used for primates and mini-pigs as anesthetics, and avertin (250–400 mg/kg, IV) was used for mice. In the case of euthanized pig was injection of ketamine (5 mg/kg, IV) followed by KCL injection (75–150 mg / kg) and mice were 99% purity CO_2_ gas according to the NIH guidelines.

### Chemicals

All chemicals and reagents used in the present study were purchased from Sigma-Aldrich Chemical Co. (St. Louis, MO, USA) unless otherwise noted.

### Isolation and culture of various species skin tissues and primary cells

Primary fibroblasts were isolated from ear skin and kidney in KSP mini-pig, strain developed by FARRC [17] and cynomolgus monkey on day 1 to 7 after birth. Bovine ear skin fibroblasts were obtained from local slaughterhouse cow ear. Neonatal tissues (ear skin and kidney) and adult tissues (bovine ear skin) were chopped using surgical scissors and then seeded into 75 cm^2^ T-flask. C57BL/6N mouse embryonic fibroblasts (MEFs) were isolated at day 13.5 (E13.5). Used HEK 239 cells were purchased from ATCC. Primary cells and cell lines were cultured in high-glucose Dulbecco's Modified Eagle's Medium (DMEM; 11995, ThermoFisher) supplemented with 10% fetal bovine serum (FBS; 16000, ThermoFisher) and 1% penicillin/streptomycin (15140, ThermoFisher). Exceptionally, 4 ng/ml basic fibroblast growth factor (bFGF; 233-FB-025, R&D systems) is added to kidney tissue derived cells.

### Oocyte collection and in vitro maturation (IVM)

Bovine and porcine ovaries were obtained from a local slaughterhouse and transported to the laboratory in 0.9% saline supplemented with 75 μg/mL potassium penicillin G and 50 μg/ml streptomycin sulfate and maintained at 25–30°C. To recovered mouse embryos, C57BL/6J female mice were super-ovulated with 5 IU of pregnant mare serum gonadotropin (PMSG; Sigma-Aldrich), followed by 5 IU of human chorionic gonadotropin (hCG; Sigma- Aldrich) 48 h apart and mated with male C57BL/6J mice. Additional details were performed as previously described [18, 19].

### Universal primer design

In order to effectively detect the ER stress in various animal species, u/s/tXBP1 primer were designed to relative the 26 bp of the XBP1 removed by IRE1 gene. Primer was designed by comparing the sequence of human, monkey, cow, pig and mouse, using of the base sequence that are well conserved XBP1 gene. For the normalization on the gene expression, a primer was produced for the GAPDH, HPRT, H2afz, 18S rRNA, ActB genes known as housekeeping genes, and a total amount of XBP1 gene expression was used to the precise ER stress. (S1 Table)

### Stimulation of ER stress in various cells and embryos

ER stress was induced in various kinds of somatic cells and embryos used in the experiment that were exposed to tunicamycin (TM, 2 μg/mL) or thapsigargin (TG, 50 nM) for various time periods. The chemicals were provided as lyophilized powders and dissolved in DMSO (TM, TG) to give stock solutions. The respective diluents were used as a negative control in each experiment.

### Semi-quantitative PCR (semi-qPCR) and quantitative Real-time RT-PCR (qPCR)

After chemical treatment the various cells and embryos were washed three times, total RNAs and mRNAs were extracted from using the Qiagen RNeasy plus kit (Qiagen) and Dynabeads mRNA DIRECT Kit (Invitrogen) according to the manufacturer’s protocol. Additional details were performed as previously described [20].

### Statistical analyses

All experiments were repeated at least three times, and data are presented as the means ± standard error (SE). Differences in the mean percentages of blastocysts and hatching blastocysts among the treatments were compared using factorial ANOVA, followed by Duncan’s multiple range tests using sigma stat software (Systat Software Inc., San Jose, CA, USA). P-values less than 0.05 were considered statistically significant.

## Results

### Differential patterns of XBP1 mRNA expression depending on the housekeeping gene used for normalization

**Fig 1:**
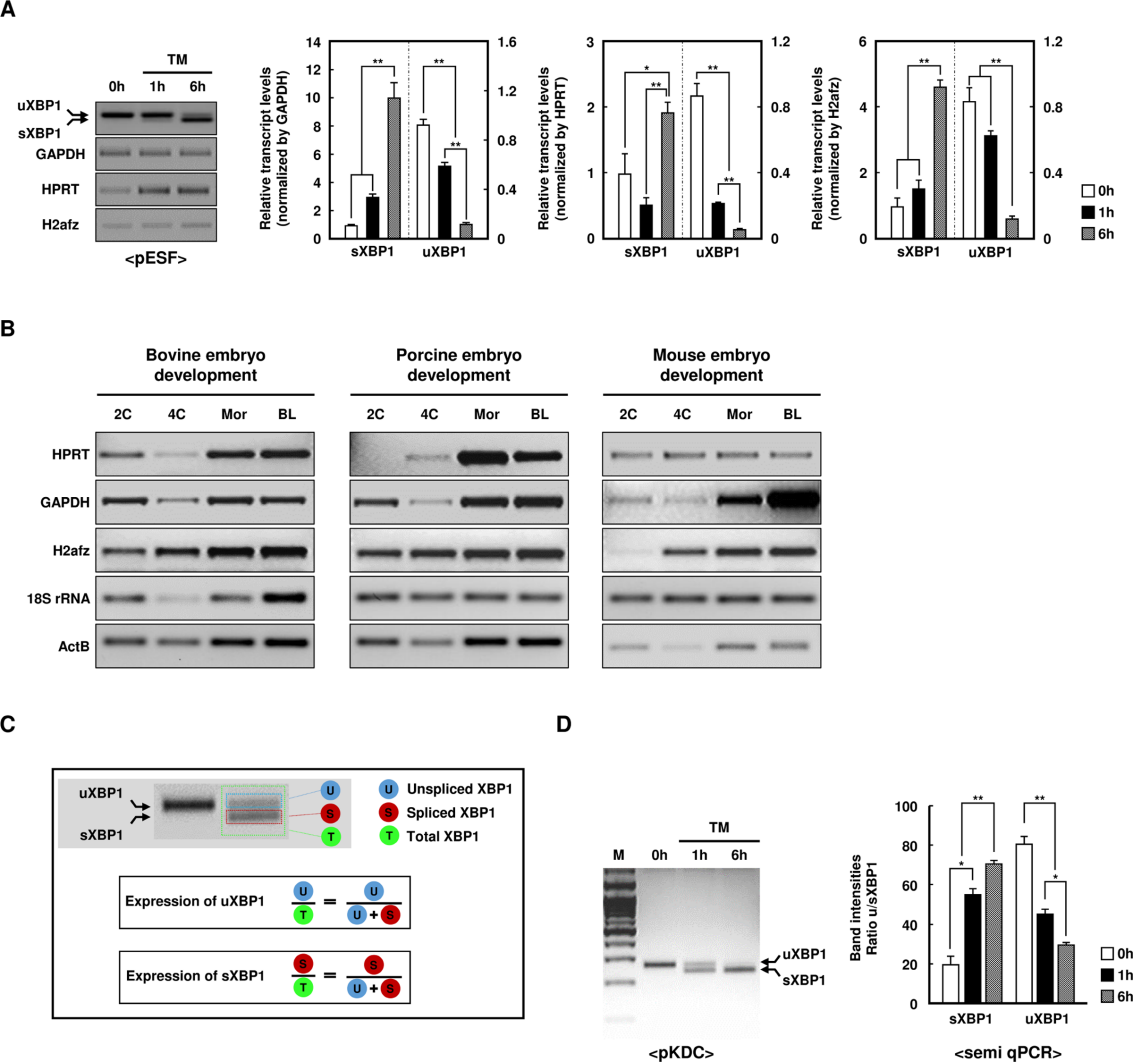
Detection of the *XBP1* gene expression using the semi-quantitative polymerase chain reaction (semi-qPCR). (A) Semi-qPCR analysis of unspliced/spliced (u/s) XBP1 and housekeeping genes (GAPDH, HPRT, or H2afz) using porcine ear skin fibroblast cells (pESF) following 0–6 h supplementation with tunicamycin at 2 μg/mL (TM; left panel). Densitometric analysis of the u/sXBP1 band intensities (right panel). GAPDH, HPRT, or H2afz was used as a normalization control (except in the first panel). Values represent the mean ± standard error (SE) (n = 3; *P < 0.05; **P < 0.01). (B) Semi-qPCR analysis of housekeeping genes (GAPDH, HPRT, H2afz, 18S rRNA, or ActB) during the early embryonic development stage (2-cell, 4-cell, morula, and blastocysts) in bovine, porcine, and mouse embryos. Three pools of 20 embryos were used for each developmental stage. (C) To measure the transcript levels of sXBP1, an indicator of endoplasmic reticulum (ER) stress, reverse transcription-PCR band intensities were densitometrically analyzed, and the levels of sXBP1 and uXBP1 relative to total XBP1 were calculated. (D) Semi-qPCR analysis of u/sXBP1 using porcine kidney-derived primary cells (pKDCs) following 6-h supplementation with TM (left panel). Densitometric analysis of the u/sXBP1 band intensity ratio (right panel). Values represent the mean ± SE (n = 3; *P < 0.05; **P < 0.01).

To quantify the level of XBP1 mRNA expression more accurately, data must be normalized to an internal control reference gene that is detected with relatively high confidence in various samples. The considerable variation in housekeeping genes caused by ER stress has hindered their systematic analysis. Here, we tested a set of candidate housekeeping genes for the normalization of XBP1 mRNA in porcine ear skin fibroblasts (pESFs) before and after tunicamycin and thapsigargin (TM and TG, respectively, ER stress inducers) treatment and examined their expression by PCR. PCR products of the sXBP1 isoform increased markedly at 6 h after treatment with TM and TG (Figs 1 and S1A). However, Fig 1A shows different patterns of XBP1 mRNA expression and variation among the housekeeping genes. Glyceraldehyde-3-phosphate dehydrogenase (GAPDH) and H2A histone family member Z (H2afz) were somewhat similarly expressed, but hypoxanthine phosphoribosyltransferase (HPRT) expression differed between non-TM and TM treatment.

To identify the most suitable reference genes, we evaluated housekeeping genes in various samples, including different developmental stages of various types of bovine, porcine, and mouse embryos. However, the expression of HPRT, GAPDH, H2afz, 18S ribosomal RNA (18S rRNA), and β-actin (ActB) exhibited significant variation depending on the state of the sample (Fig 1B). These results imply that XBP1 mRNA expression differs depending on the reference gene used for normalization. To address this problem, we and others previously suggested a tXBP1 assay [7] in which band intensities are quantified using ImageJ software and the percentage of sXBP1 is calculated by dividing the signal for sXBP1 mRNA by the sum of the signals (tXBP1) for sXBP1 and uXBP1 mRNAs (Fig 1C and 1D). Therefore, because housekeeping genes differ, the normalization of XBP1 mRNA expression by tXBP1, which is commonly detected, is required.

### Analysis of conserved regions of XBP1 mRNA using various animals and design of the universal primers

**Fig 2:**
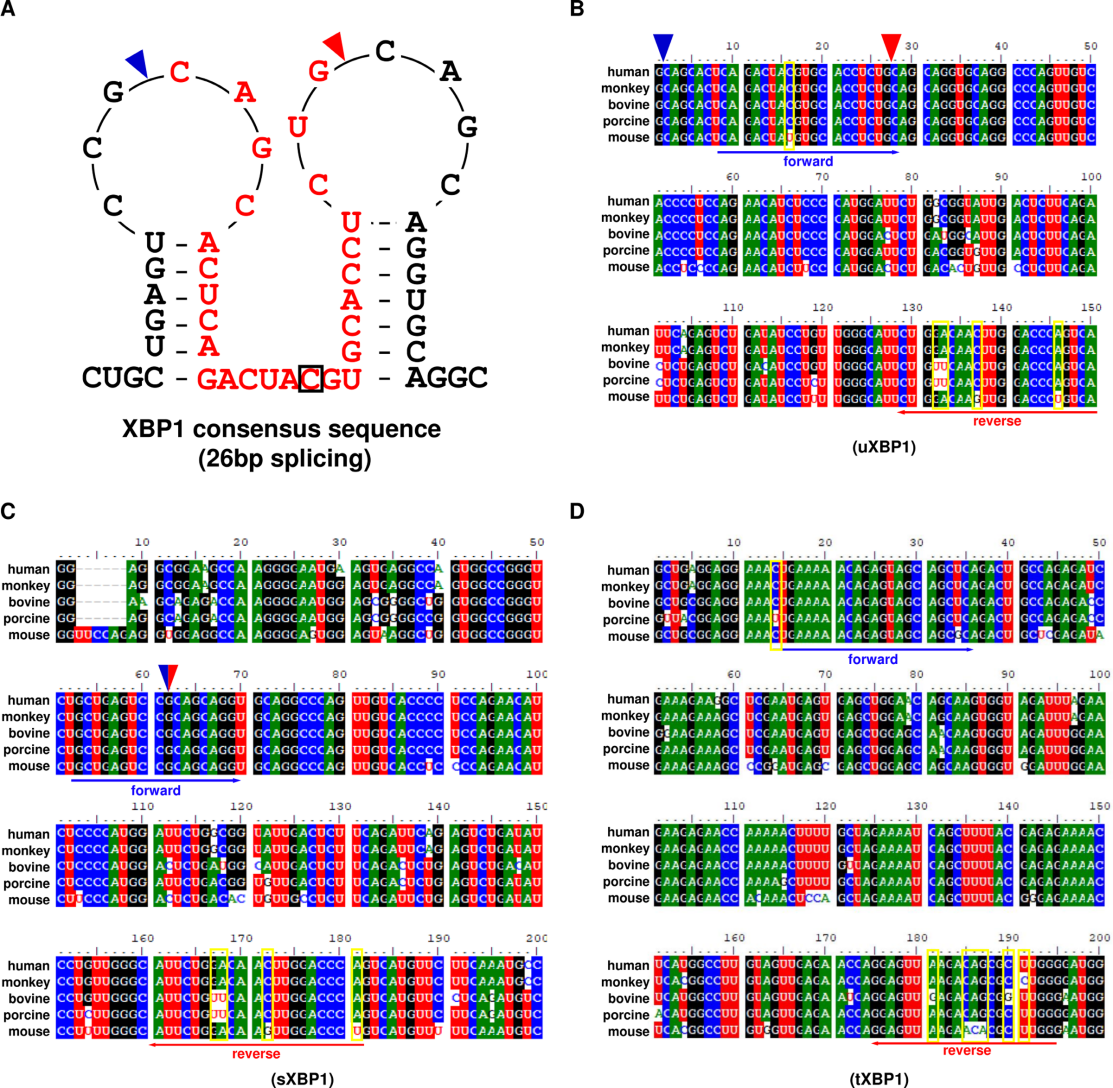
Primers targeting the u/s/total (t) XBP1-conserved sequence in various species. (A) The consensus sequence of XBP1 in human, monkey, cow, pig, and mouse. The blue triangle represents the start, and the red triangle represents the end, of the spliced 26 bp intron. The red text indicates the splicing region of XBP1. Black squares indicate the difference sequence of C to U in mice. (B–D) Alignment of the u/s/tXBP1 gene among the five species and the location of each XBP1 primer. The blue triangle indicates the start, and the red triangle indicates the end, of the spliced 26 bp intron. The blue arrow indicates the forward primer and the red arrow indicates the reverse primer.

XBP1 differs in the size of the mRNA produced in the presence or absence of ER stress, because XBP1 mRNA is spliced via the removal of a 26 bp intron from uXBP1 by IRE1α. The secondary structure of XBP1 mRNA around the splice site shares common features in various species and splice sites are located in the loop regions of each short hairpin [8]. As expected, a splice site consisting of the same base sequence was observed in the majority of animals, but was only 1 bp (C to U) in mice (Fig 2A). Here, we expanded the concept to design desirable universal real-time PCR primers for XBP1 mRNA and performed multiple sequence alignments of RNA segments adjacent to the splice site in human, monkey, bovine, porcine, and mouse samples. We found conserved positions based on 26 bp positions truncated by IRE1α and primer positions to detect uXBP1, sXBP1, and tXBP1 (Fig 2B–2D). The universal primer sets for each u/s/tXBP1 use a mixed base code and were designed to amplify product sizes of 139, 130, and 181 bp, respectively, without nonspecific amplification (S1 and S2 Tables). The specificity of the designed primers was examined by gel electrophoresis, and target amplicons were a single sharp melting curve in real-time PCR (S2 Fig). To further increase stringency, the amplification efficiencies and correlation coefficients (R^2^) of the universal primers in sXBP1, uXBP1, and tXBP1 were calculated by the slopes of the standard curves. The real-time PCR amplification efficiencies for each u/s/tXBP1 ranged from 92.8 to 115.1 and the correlation coefficients ranged from 0.981 to 1.000 (S3 Table). Therefore, these primers can be used for real-time PCR analysis. To our knowledge, this is the first time that XBP1 activity was easily measured by real-time PCR analysis using a universal primer method that is measurable in various animals.

### Quantitative comparison of tXBP1 and housekeeping genes for XBP1 activity

**Fig 3:**
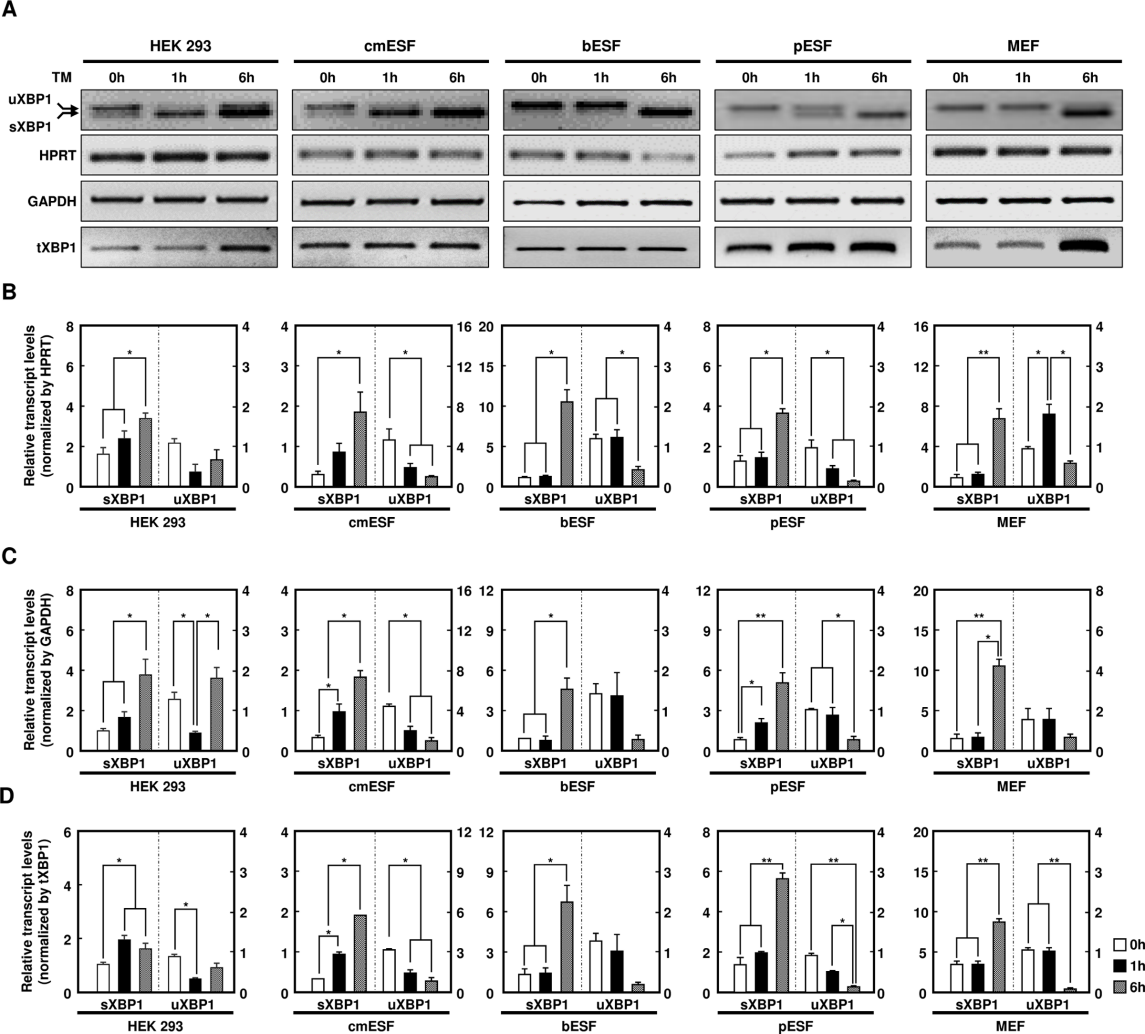
Analyses of XBP1 expression in various species using semi-qPCR. (A) Semi-qPCR analysis of u/s/tXBP1 and housekeeping genes (HPRT or GAPDH) using human embryonic kidney 293 (HEK293), cynomolgus monkey ESF (cmESF), bovine ESF (bESF), porcine ESF (pESF), and mouse embryonic fibroblast (MEF) cells following 0, 1, 6 h supplementation with tunicamycin. (B–D) Densitometric analysis of u/sXBP1 band intensities. The tXBP1 employed for quantification was used as a universal primer, 181 bp in size, as shown in Fig 2D. HPRT, GAPDH, or tXBP1 was used as a normalization control. Values represent the mean ± SE (n = 3; *P < 0.05; **P < 0.01).

Next, we normalized housekeeping genes or tXBP1 to measure the precise activity of XBP1 following TM treatment. We observed increased sXBP1 mRNA levels following TM treatment in human, monkey, bovine, porcine, and mouse cells, and quantification was performed with HPRT, GAPDH, and tXBP1. As shown in Fig 3, uXBP1 and sXBP1 expression patterns were similar in all species (Fig 3A), but different results were obtained when quantifying the band intensities of HPRT, GAPDH, and tXBP1 (Fig 3B–3D). The quantitative results in fibroblast cells, such as human embryonic kidney 293 (HEK293) cells, bovine ESFs (bESFs), pESFs, and mouse embryonic fibroblasts (MEFs), showed significant differences. This finding indicates that XBP1 activity may differ according to the experimental conditions used and depending on the housekeeping gene or the state of the sample. For this reason, it is necessary to quantify XBP1 using tXBP1 rather than a housekeeping gene to obtain more accurate and reliable results.

### Validation of XBP1 mRNA using universal real-time PCR primers

**Fig 4:**
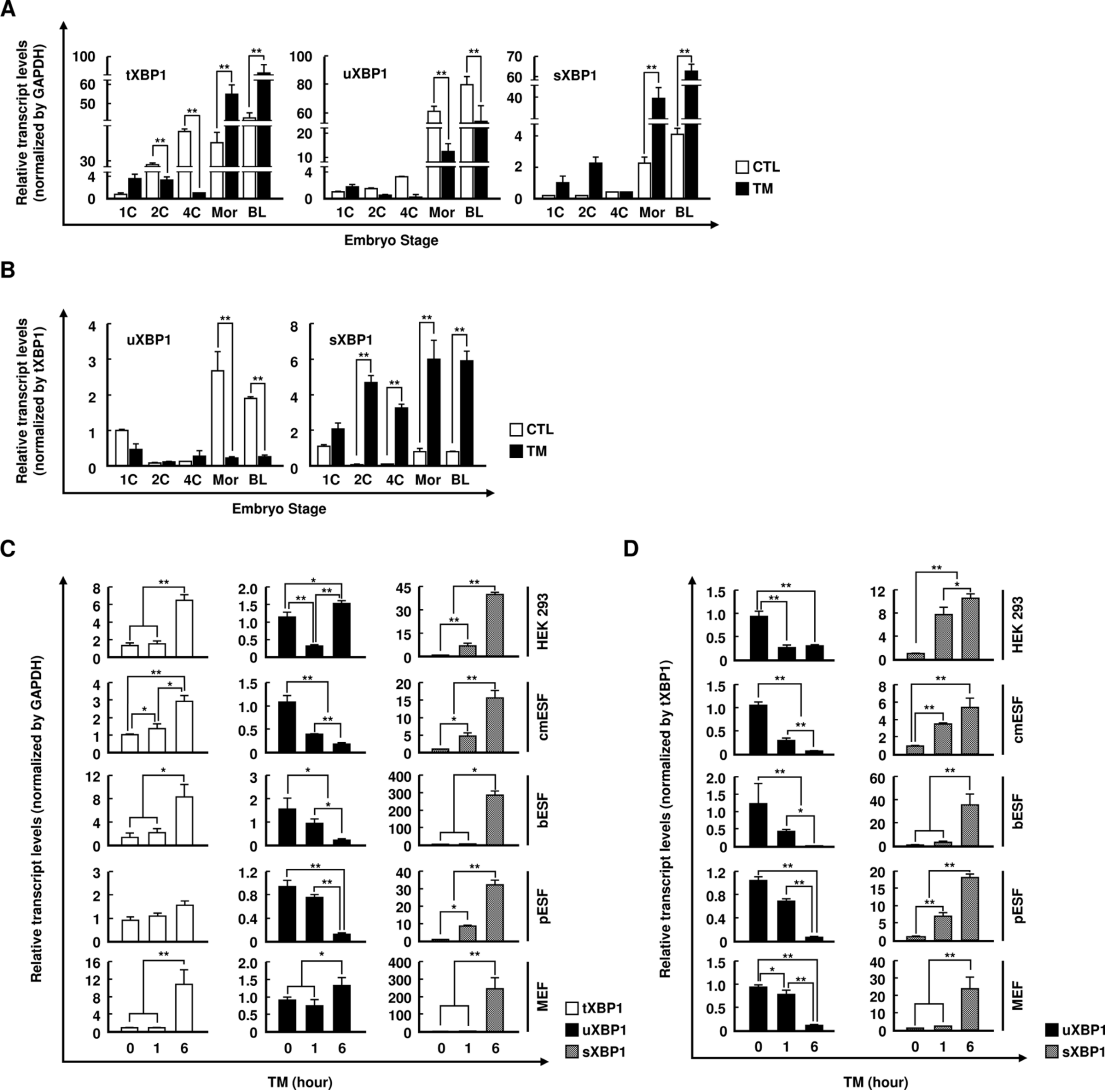
Examining the expression of u/s/tXBP1 during early embryonic development in various species using qPCR. (A and B) qPCR analyses of the relative abundances of u/s/tXBP1 in porcine embryos at early developmental stages (1-cell, 2-cell, 4-cell, molura, and blastocyst) in the absence and presence of tunicamycin. GAPDH or tXBP1 was used as a normalization control. Values represent the mean ± SE (n = 3; **P < 0.01). (C and D) qPCR analyses of the relative abundances of u/s/tXBP1 in HEK293, cmESF, bESF, pESF, and MEF cells cultured for 0, 1, 6 h with TM. GAPDH or tXBP1 was used as a normalization control. Values represent the mean ± SE (n = 3; *P < 0.05; **P < 0.01).

To demonstrate the efficacy and applicability of universal primers capable of measuring the activity of XBP1 in a variety of species, we confirmed our results using real-time PCR. When porcine embryos were supplemented with TM for 1 h during *in vitro* embryonic development, the expression of sXBP1 was significantly increased when using the universal real-time PCR primers in 1-cell, 2-cell, 4-cell, morula, and blastocyst stages. We also confirmed that tXBP1 increased when normalized to GAPDH in this process (Fig 4A). However, these results differed when normalized to tXBP1. In particular, we observed significant differences in sXBP1 levels between 2-cell and 4-cell stages, which did not differ when normalized to GAPDH following TM treatment (Fig 4A and 4B). In accordance with these results, the differences in XBP1 mRNA expression following TM or TG treatment in various species also differed according to whether the results were normalized to GAPDH, 18s rRNA, ActB, or tXBP1 (Figs 4C and 4D and S1B, S1C and S3). This indicates that correct selection of the reference gene is important for proper analysis of real-time PCR results. Taken together, to reduce the variability in gene expression levels related to the use of housekeeping genes, a common reference gene should be used to measure and reduce error resulting from variation among samples.

## Discussion

ER stress can be detected in many ways (e.g., by confirming protein expression via introducing a vector construct into cells [21], and producing an animal model to directly confirm ER stress *in vivo* [12]). However, because it can be difficult to detect ER stress using these methods, we aimed to improve the method for quantifying ER stress using PCR assays. First, we designed a universal primer with conserved region analysis of XBP1 mRNA in various experimental animals, to allow investigators in various fields to measure ER stress. Notably, the *XBP1* gene of human, monkey, cow, pig, and mouse was found to be conserved at the 26 bp position, and to be cleaved by IRE1α in the primer position used to detect u/s/tXBP1. In the present study, we designed primers that spanned the 26 bp intron junction or were expressed inside the 26 bp intron of XBP1 mRNA, to distinguish between sXBP1 and uXBP1 mRNAs (Fig 2). Although the concept of the u/sXBP1 primer set is similar to that described in a previous study [11], that study was limited because it was concerned only humans. Accordingly, we designed and experimented with a set of universal primers that could measure the activity of XBP1 in the five most commonly used mammalian models. Moreover, to increase the reliability of the universal primer, the slope, intercept, amplification efficiency, and correlation coefficient (R^2^) of the primer pair were calculated as described previously (S3 Table and S2 Fig) [22, 23].

The methods for evaluating the increase in sXBP1 was as follows. The first method was semi-qPCR, using an XBP1-specific primer capable of detecting both sXBP1 (263 bp) and uXBP1 (289 bp) by normalizing the ratio of the mRNA levels of sXBP1 to uXBP1 with a housekeeping gene such as *GAPDH* or actin [24, 25]. This method was based on the PCR results obtained using a single primer set, but a disadvantage is that a high agarose concentration [2% (w/v)] must be used, because the bands of sXBP1 and uXBP1 are very close to each other and sufficient separation time is required. The second method used PCR and the restriction endonuclease, *Pst* I. XBP1 mRNA contains a 26 bp intron, in turn containing the *Pst* I restriction enzyme, whereas sXBP1 mRNA does not contain this Pst I site because the intron has been removed. Thus, *Pst* I digestion cleaved uXBP1 into two small fragments, while sXBP1 migrated as one fragment without being cleaved by *Pst* I [10]. This method allows for better discrimination between sXBP1 and uXBP1, but it requires additional restriction enzyme treatment after PCR and the results are affected by the restriction enzyme. The next method involved PCR performed using specific primers of uXBP1 and sXBP1, followed by loading the PCR products together. Although it was easier to identify sXBP1 because it was larger than 26 bp, a limitation is that interpretation of the results can differ depending on the PCR conditions. Despite the existence of these various measurement methods, differences between the two XBP1 bands must be visualized by gel electrophoresis, and the data proved difficult to interpret. However, this study showed that using the qPCR-based method improved the efficiency and accuracy of the XBP1 activity measurements.

qPCR is a very efficient and accurate method to assess the transcription levels of candidate genes, particularly in small samples and preimplantation embryos [26]. However, many studies have provided convincing evidence that the level of transcription of housekeeping genes differs according to the developmental stage and other experimental conditions [27, 28]. For determination of the best candidate reference gene, the following statistical five algorithms were used: NormFinder, geNorm, BestKeeper, ΔCT, and coefficient of variation [29]. However, normalization results, which cannot be solved by the aforementioned algorithms, vary depending on the housekeeping gene used (e.g., GAPDH, HPRT), the expression of which varies during embryonic stem cell differentiation and according to the stage of embryogenesis in specific tissues [14, 16, 30]. Consistent with previous studies, we found that housekeeping genes have different patterns of expression depending on the species and development stage, as shown in Fig 1B. We found that selection of an inappropriate housekeeping gene can give rise to misinterpretation of the data. Our results also confirmed that sXBP1 levels were dependent on the housekeeping genes used for normalization (Figs 1 and 3). Thus, until further improvements in analysis methods are realized, we suggest the use of tXBP1 as an adequate endogenous control for normalization of sXBP1 and uXBP1 expression, for more accurate detection regardless of the tissue type or developmental stage. Although the expression of the *XBP1* gene differed among various conditions, the expression levels of uXBP1 and sXBP1 may be measured more reliably than those of other housekeeping genes by using tXBP1, which has been designed as a component common to both uXBP1 and sXBP1. In 2018, Stork and colleagues also reported the use of designed qPCR primer pairs to measure total, spliced, and unspliced Xist transcripts, and normalized the spliced and unspliced Xist to total Xist [31]. In addition, several studies have shown that quantification using the total gene transcript is more accurate for quantifying housekeeping genes [32, 33]. In conclusion, the results of the present study validated our methodological concept and demonstrated that our method was sensitive enough to detect even small changes in the ratio of sXBP1 to uXBP1, in various sample types.

## Supporting information

S1 Fig Examining the expression of XBP1 under ER stress conditions. (A) Semi-qPCR analysis of u/sXBP1 using pESF cells following 0–6 h supplementation with TG. (B and C) qPCR analyses of the relative abundances of u/s/tXBP1 in HEK293, cmESF, bESF, pESF, and MEF cells cultured for 0–6 h with TG. GAPDH or tXBP1 was used as a normalization control. Values represent the mean ± SE (n = 3; *P < 0.05, **P < 0.01).
(TIF)

S2 Fig Melting curves and amplification plots for u/s/tXBP1 and GAPDH. (A and B) Melting curves and amplification plots were obtained using universal primers targeting u/s/tXBP1 and housekeeping genes in HEK 293, cmESF, pESF, bESF, and MEF cells.
(TIF)

S3 Fig Expression of XBP1 normalized using various housekeeping genes. (A and B) qPCR analyses of the relative abundances of u/s/tXBP1 in HEK293, cmESF, bESF, pESF, and MEF cells cultured for 0–6 h with TM. 18S rRNA or ActB was used as a normalization control. Values represent the mean ± SE (n = 3, *P < 0.05, **P < 0.01).
(TIF)

S1 Table Primer sequences used for semi-qPCR and qPCR. (TIF)

S2 Table Universal primer sequences and qPCR. (TIF)

S3 Table PCR efficiency for u/s/tXBP1 primer pairs. (TIF)
