# Supplementary figures and images for: Real-time PCR quantification of spliced X-box binding protein 1 (XBP1) using a universal primer method
Source: PLoS One. 2019 Jul 22;14(7):e0219978. doi: 10.1371/journal.pone.0219978 (PMC6645673; doi:10.1371/journal.pone.0219978)

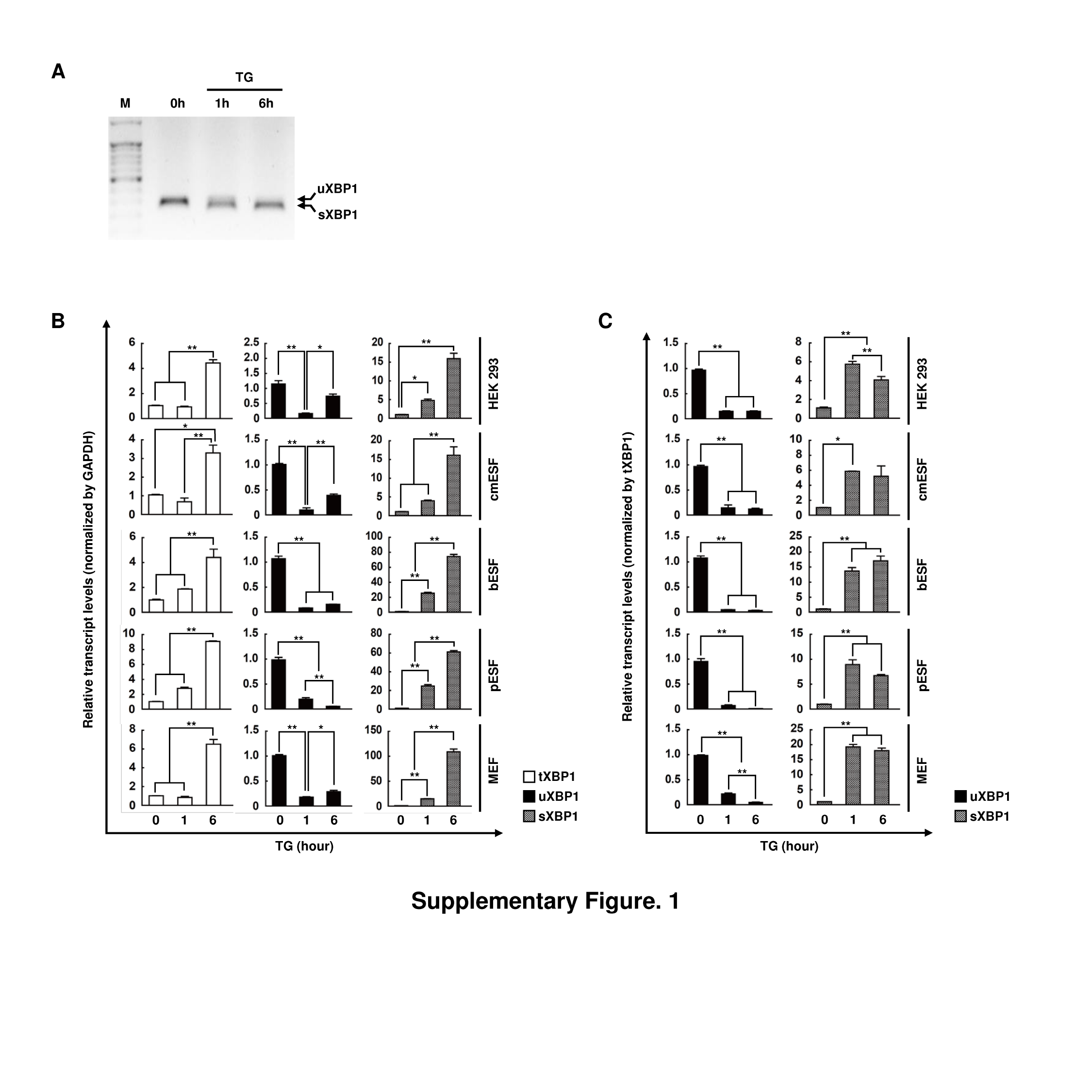

Supplement: S1 Fig — (A) Semi-qPCR analysis of u/sXBP1 using pESF cells following 0–6 h supplementation with TG. (B and C) qPCR analyses of the relative abundances of u/s/tXBP1 in HEK293, cmESF, bESF, pESF, and MEF cells cultured for 0–6 h with TG. GAPDH or tXBP1 was used as a normalization control. Values represent the mean ± SE (n = 3; *P < 0.05, **P < 0.01). (TIF) [file pone.0219978.s001.tif]

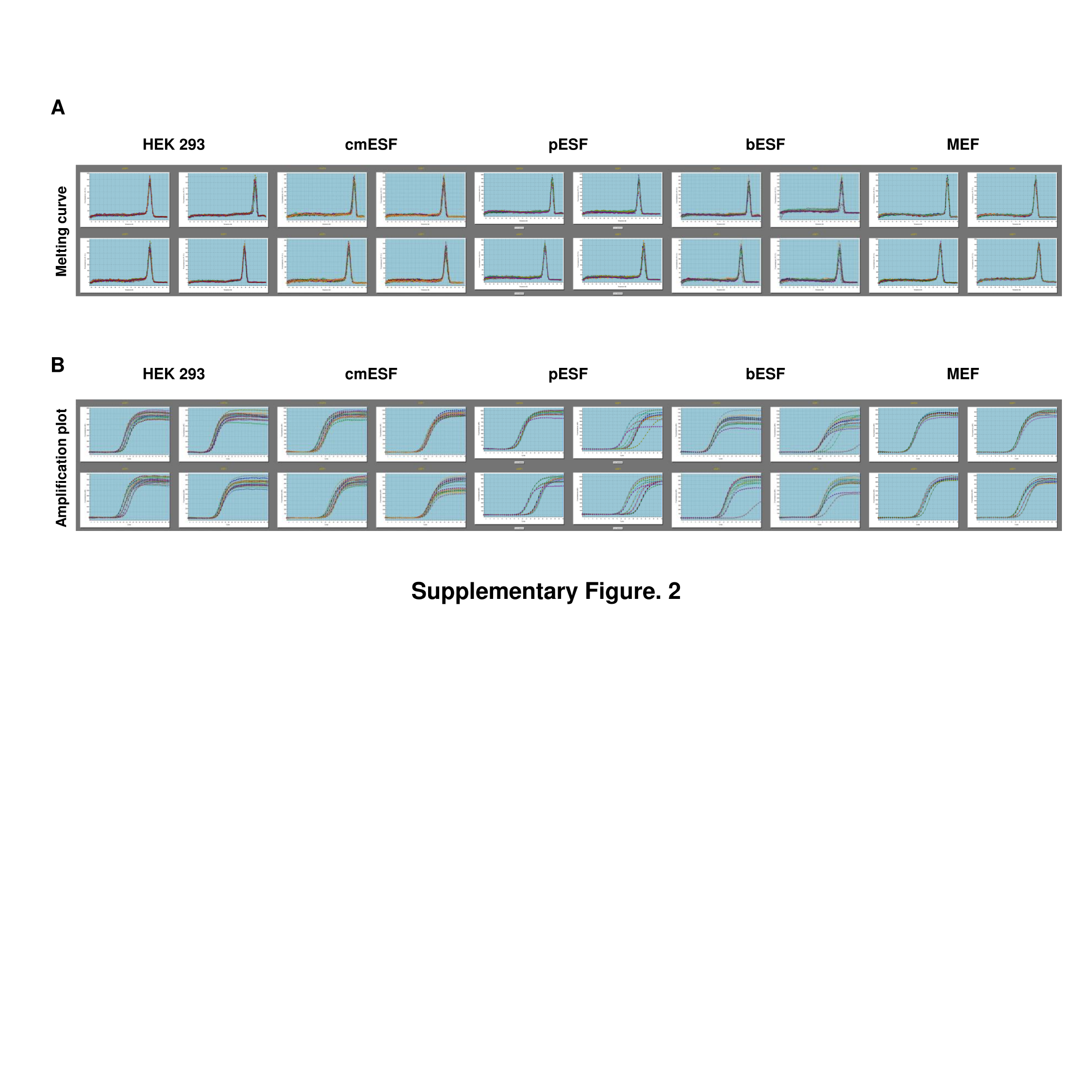

Supplement: S2 Fig — (A and B) Melting curves and amplification plots were obtained using universal primers targeting u/s/tXBP1 and housekeeping genes in HEK 293, cmESF, pESF, bESF, and MEF cells. (TIF) [file pone.0219978.s002.tif]

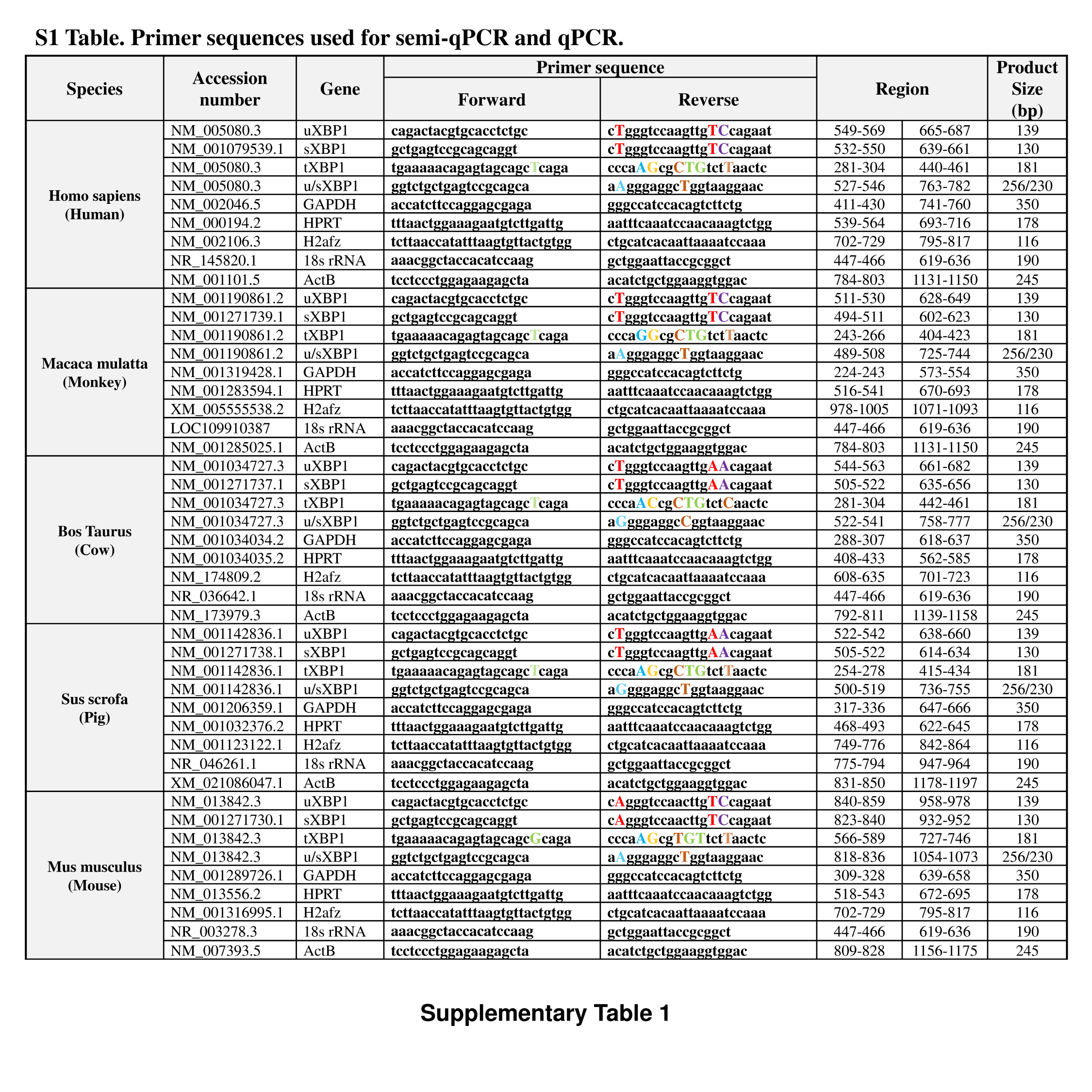

Supplement: S1 Table — (TIF) [file pone.0219978.s004.tif]

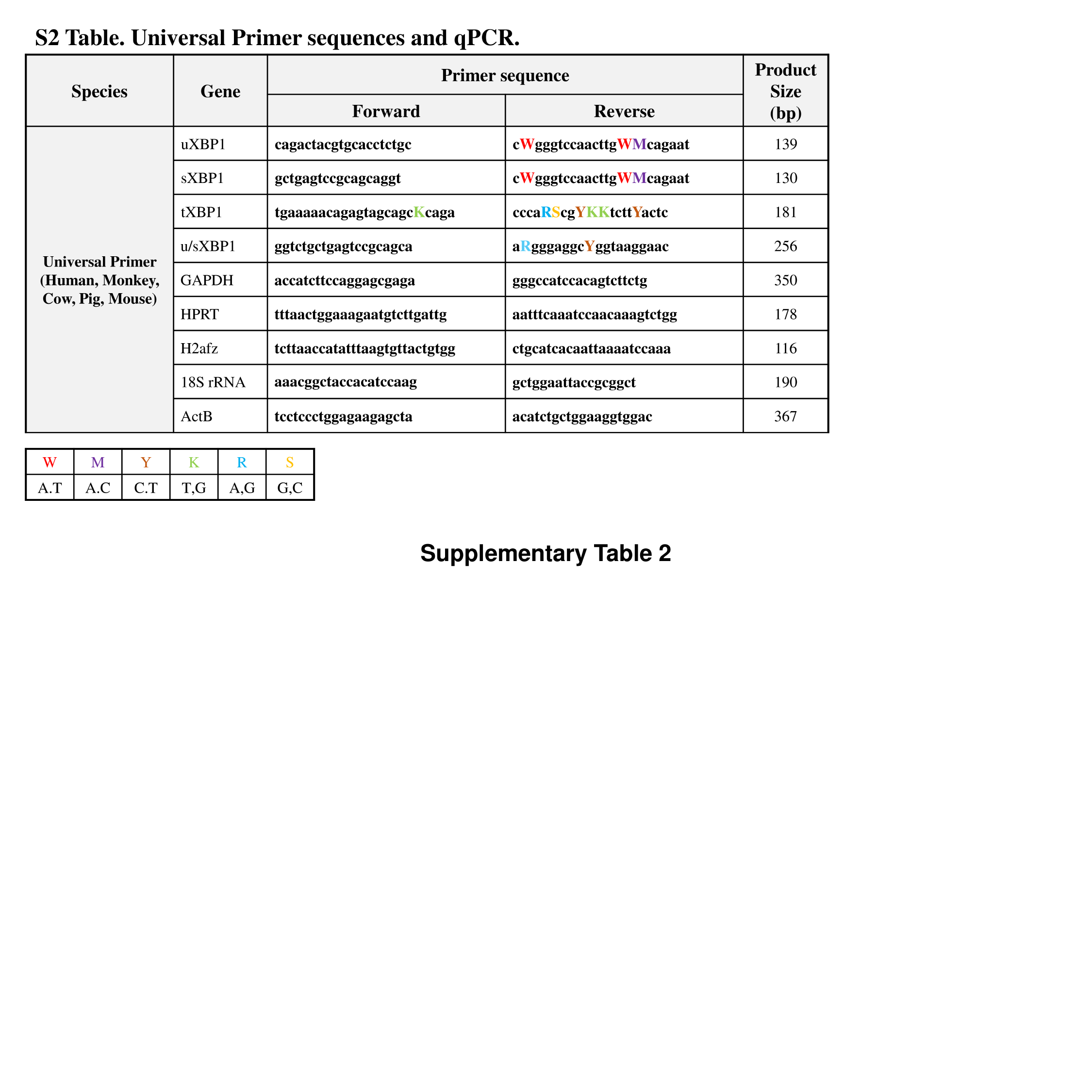

Supplement: S2 Table — (TIF) [file pone.0219978.s005.tif]

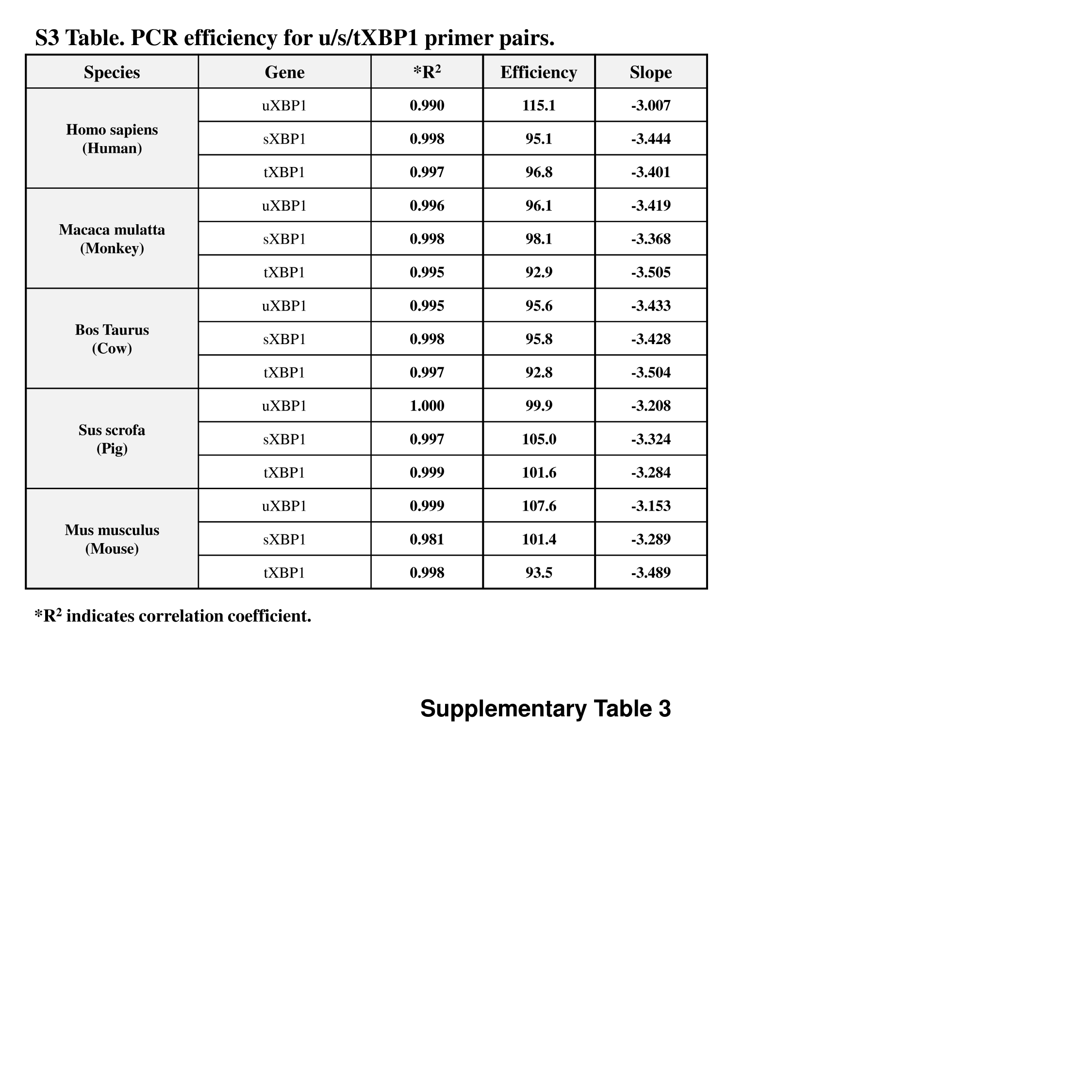

Supplement: S3 Table — (TIF) [file pone.0219978.s006.tif]
